# Superior mesenteric artery embolism associated with Cisplatin-induced aortic thrombosis

**DOI:** 10.1259/bjrcr.20220149

**Published:** 2023-09-12

**Authors:** Ryo Aoki, Shingo Kato, Kento Nakajima, Jun Sakai, Kenichi Yoshida, Hidenori Masui, Shin Ikeda, Jun Yoshigi, Daisuke Utsunomiya

**Affiliations:** 1 Department of Diagnostic Radiology, Yokohama City University Graduate School of Medicine, Yokohama, Japan; 2 Department of Surgery, Yokosuka Kyosai Hospital, Yokosuka, Japan; 3 Department of Radiology, Yokosuka Kyosai Hospital, Yokosuka, Japan

## Abstract

Cardiovascular complications of cancer therapy are among the most important factors affecting cancer prognosis. Cisplatin-induced aortic thrombosis is rare but can be life-threatening in the event of peripheral embolism. In this report, we describe a case of superior mesenteric artery (SMA) embolism associated with cisplatin-induced aortic thrombosis. A 66-year-old male, diagnosed with esophageal cancer, initiated systemic chemotherapy with a regimen consisting of 5-fluorouracil and cisplatin, combined with radiotherapy. After 7 days of chemoradiotherapy, the patient developed a floating thrombus in the ascending aorta and an SMA embolism; chemoradiotherapy was then discontinued. Laparoscopy revealed an ischemic small intestine that required resection; intravenous unfractionated heparin was initiated 3 days after. Computed tomography showed disappearance of the floating aortic thrombus and reduce SMA thrombus size. Early detection of cisplatin-induced aortic thrombosis may prevent fatal outcomes in symptomatic peripheral embolisms, such as SMA embolism, considering anticoagulation, and discontinuation of cisplatin-based chemotherapy may cause resolution of thrombus events.

## Introduction

Cardiovascular complications of cancer therapy are one of the most important factors affecting the prognosis of patients with cancer. Cisplatin-induced aortic thrombosis is rare but can be life-threatening in the event of peripheral embolism. This report describes a case of superior mesenteric artery (SMA) embolism associated with cisplatin-induced aortic thrombosis.

## Case report

**Figure 1.**
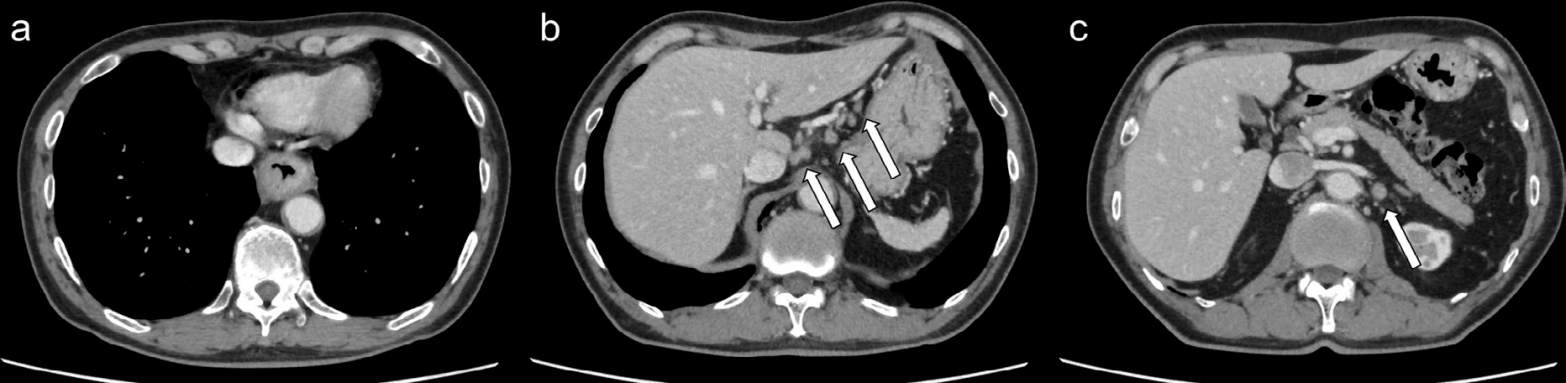
(**a**) Axial contrast-enhanced computed tomography shows the primary lower thoracic esophageal cancer. (**b, c**) Axial contrast-enhanced computed tomography shows lymph node metastasis involving the celiac, left gastric and para-aortic regions (arrows).

**Figure 2.**
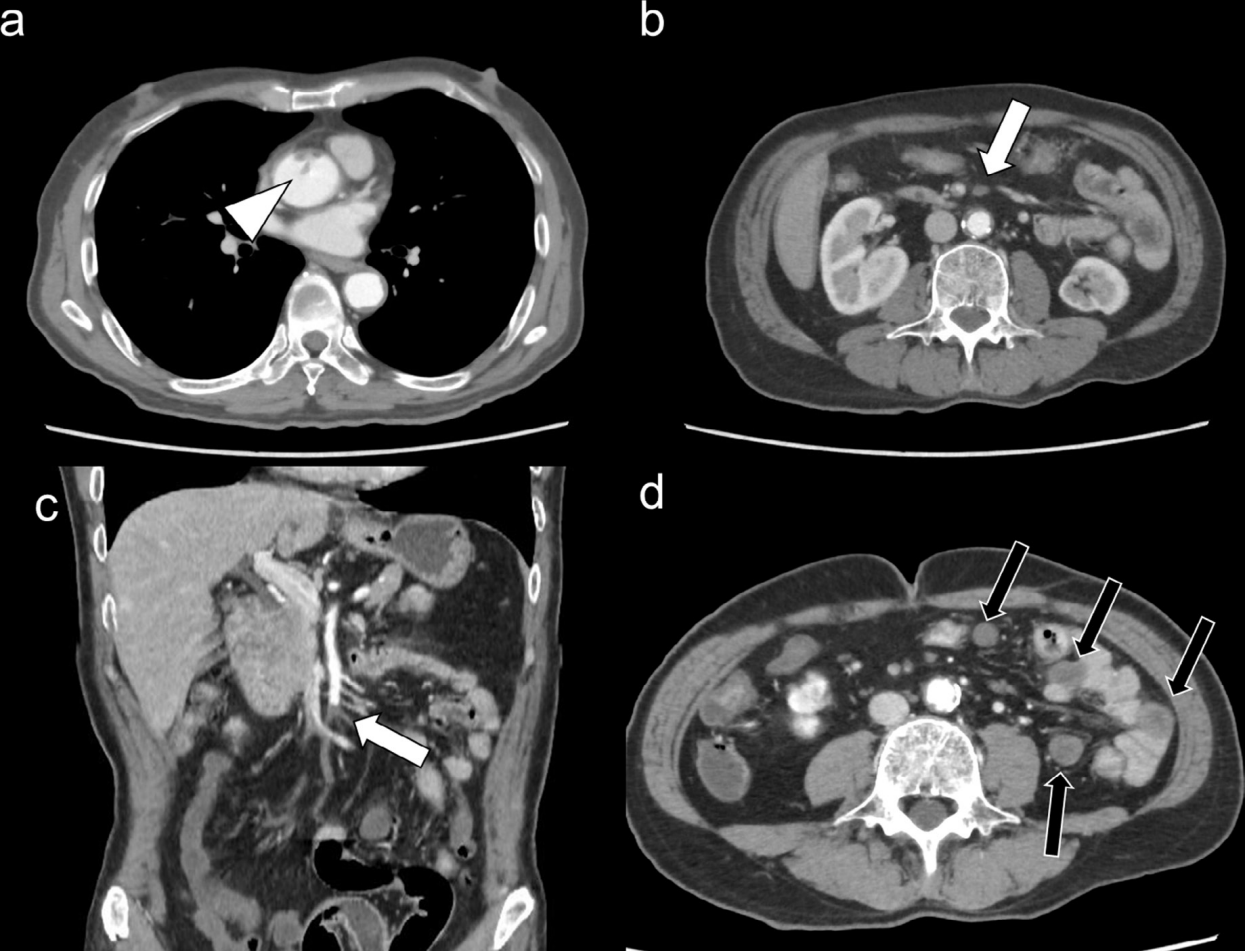
Contrast-enhanced computed tomography shows an aortic thrombus in the ascending aorta (**a**) and SMA embolism (b: axial view, c: coronal view). The arrowhead indicates a floating thrombus and white arrows indicate SMA embolism. Contrast-enhanced computed tomography also showed decreased contrast enhancement in the small intestine (d: black arrows).

**Figure 3.**
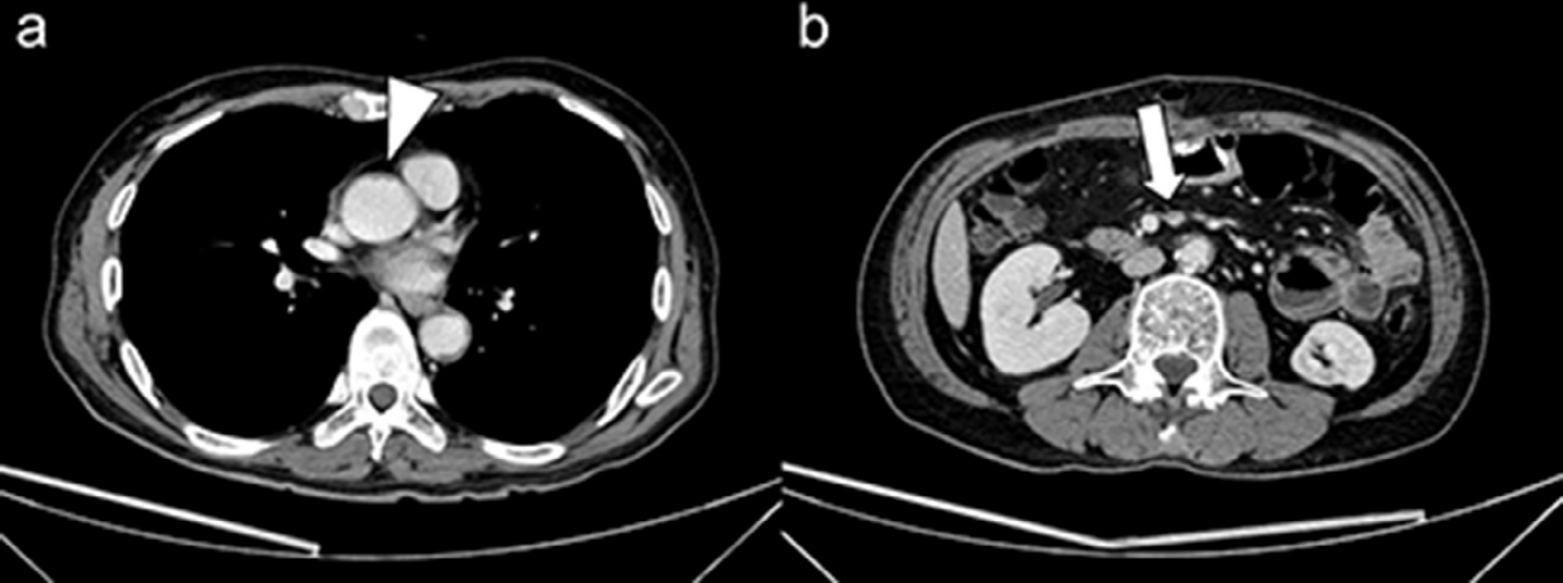
Contrast-enhanced CT shows resolution of the floating aortic thrombus (**a**) and reduction in the size of the SMA thrombus (**b**). Arrowhead indicates resolution of the aortic floating thrombus, and the arrow indicates reduction in the size of the SMA thrombus.

A 66-year-old male was diagnosed with lower thoracic esophageal cancer cT3N2M1 (Figure 1), cStage IVB (according to the eighth edition of TNM classification of the Union for International Cancer Control [UICC-TNM eighth edition]). Systemic chemotherapy was initiated with a regimen consisting of 5-fluorouracil (5-FU) and cisplatin, combined with radiotherapy (total 60 Gy/ 30 Fr). After 7 days of chemoradiotherapy, the patient developed acute abdominal pain. Therefore, contrast-enhanced chest-abdomen computed tomography (CT) was performed to identify the cause of the acute abdominal pain and evaluate esophageal cancer status. Contrast-enhanced CT showed a floating thrombus in the ascending aorta and an SMA embolism, which did not exist prior to chemoradiotherapy (Figure 2). Contrast-enhanced CT also showed decreased contrast enhancement in the small intestine, suggesting intestinal ischemia. Additionally, contrast-enhanced CT showed no ischemia in other organs. There were also no clinical symptoms suggestive of emboli in the lower extremities. Laboratory results were as follows: hemoglobin level, 14.3 g dl^−1^; hematocrit, 43.5%; platelet count, 31.0 × 10^4^/ µL; fibrinogen level, 593 mg dl^−1^; prothrombin time, 9.5 s; activated partial thromboplastin time, 25.8 s; lactate dehydrogenase, 232 U l^−1^; creatine kinase, 23 U l^−1^; C-reactive protein, 3.13 mg dl^−1^; pH, 7.415; pCO_2_, 35.4 mmHg; HCO_3_, 22.3 mmol l^−1^; base excess, −1.2 mmol l^−1^; and lactate, 2.9 mmol l^−1^; increased D-dimer levels from less than 0.4  µg ml^−1^ before chemoradiotherapy to 5.5 µg ml^−1^. Polymerase chain reaction results were negative for coronavirus disease-2019 (COVID-19). Chemoradiotherapy was discontinued. Diagnostic laparoscopy revealed an ischemic small intestine requiring bowel resection; subsequently, the procedure was converted to an open partial resection of the small intestine and intravenous unfractionated heparin was administered 3 days postoperatively. Furthermore, 8 days after the onset of SMA embolism, CT showed disappearance of the floating aortic thrombus and reduced SMA thrombus size (Figure 3). Chemotherapy resumed using a nivolumab regimen 2 months after SMA embolism onset, and thromboembolic events did not recur.

## Discussion

Cisplatin-based chemotherapy induces acute and transient endothelial dysfunction, dyslipidemia, and hyperglycemia during early phases of treatment.^1^ Owing to these effects, patients receiving cisplatin-based chemotherapy are at substantial risk for venous and arterial thromboembolic events.^2^ Although cisplatin-induced aortic thrombosis is rare, it can cause fatal embolisms. Thromboembolisms related to cisplatin-induced aortic thrombosis have been reported in the brain and other organs.^3^ Here, we have described a rare case of SMA embolism associated with cisplatin-induced aortic thrombosis, which to the best of our knowledge has not yet been reported.

Acute mesenteric ischemia is a life-threatening disease classified by arterial occlusion (both embolic and thrombotic), venous occlusion, nonocclusive mesenteric ischemia, and strangulating obstruction.^4^ SMA embolism is an arterial occlusive disease caused by atrial fibrillation or other arrhythmias, infection (*i.e.,* COVID-19),^5^ hematologic disorders (*i.e.,* antiphospholipid syndrome), and drugs (*i.e.,* cisplatin). Anticoagulation is the first-choice treatment in such cases, unless contraindicated. Laparoscopic surgery may be considered in patients who may have intestinal necrosis or peritonitis, while endovascular revascularization is the preferred initial intervention if the intestinal ischemia appears reversible.

Several drugs, including cisplatin and bevacizumab, have been implicated in chemotherapy-induced aortic thrombosis.^6^ Among these drugs, cisplatin carries a substantial risk of venous and arterial thromboembolic events, such as deep vein thrombosis, pulmonary embolism, arterial thrombosis, and cerebrovascular events.^2^ The incidence of venous and arterial thromboembolic events is reported to be 18.1% during administration period, or within 4 weeks of cisplatin-based chemotherapy completion.^2^ While pulmonary thromboembolism and deep vein thrombosis occur frequently, cisplatin-induced aortic thrombosis is rare and only a few cases have been reported.^7^ Nevertheless, we posit that the clinical importance of aortic thrombosis complications may have been underestimated considering cisplatin-induced aortic thrombosis is often asymptomatic and incidentally found on follow-up CT.^7^ Early detection of aortic thrombosis may prevent fatal outcomes in symptomatic peripheral embolisms, such as SMA embolism, given anticoagulation and discontinuation of cisplatin-based chemotherapy can cause resolution of thrombosis. Careful interpretation of follow-up CT scans, focusing on cardiovascular events and regular monitoring of D-dimer levels before and during chemotherapy, may contribute to the early detection of asymptomatic aortic thrombosis. Prophylactic oral anticoagulant use during chemotherapy may also be considered in patients at high risk for thromboembolic events,^8^ although no clear indication criteria exist, and further investigation is warranted.

Although the pathogenesis of cisplatin-induced aortic thrombosis remains unclear, Virchow’s triad refers to the factors promoting thrombosis, that is, hypercoagulability, endothelial injury/dysfunction, and stasis of blood flow. While it is generally used to assess the cause of thrombus in veins, Virchow’s triad may also be partially related to arterial thrombus formation.^9^ Cisplatin-based chemotherapy induces acute and transient endothelial dysfunction, dyslipidemia, and hyperglycemia during the early phases of treatment.^1^ Furthermore, cisplatin-based chemotherapy may cause aortitis^10^ and may lead to endothelial injury/dysfunction. These mechanisms infer development of various thromboembolic events including aortic thrombosis. However, one of the limitations of cisplatin-induced aortic thrombosis detection is the difficulty in obtaining diagnostic evidence, as it is diagnosed by exclusion of other causes. There are no reports in the literature suggesting that 5-FU or radiotherapy can induce aortic thrombosis. Therefore, it is likely that the aortic thrombosis in this patient was caused by cisplatin chemotherapy. However, the ascending aorta is also affected by radiotherapy, and endothelial damage from radiotherapy could have triggered aortic thrombosis in the ascending aorta.

## Learning points

- Early detection of aortic thrombosis may prevent fatal outcomes in symptomatic peripheral embolisms.
- Careful interpretation of the follow-up computed tomography findings, focusing on the aorta and regular monitoring of D-dimer levels before and during chemotherapy, may contribute to the early detection of asymptomatic aortic thrombosis.
